# Synthesis, crystal structure and Hirshfeld surface analysis of 4-{(1*E*)-1-[(car­bamo­thioyl­amino)­imino]­eth­yl}phenyl propano­ate

**DOI:** 10.1107/S2056989024003177

**Published:** 2024-04-18

**Authors:** Sundarasamy Madhan, MohamedHanifa NizamMohideen, Vijayan Viswanathan, Mani Karthik Ananth, Srinivasan Narasimhan

**Affiliations:** a Department of Physics, The New College, Chennai 600 014, University of Madras, Tamil Nadu, India; b Department of Biophysics, All India Institute of Medical Science, New Delhi 110029, India; c Department of Food Quality & Safety, Institute for Postharvest and Food Sciences, Volcani Center, ARO, Rishon LeZion 7528809, Israel; d Department of Chemistry, Asthagiri Herbal Research Foundation, Perungudi Industrial Estate, Chennai 600 096, Tamilnadu, India; Vienna University of Technology, Austria

**Keywords:** crystal structure, thio­semicarbazone, propionate, hydrogen bonding, Hirshfeld surface analysis, two-dimensional fingerprint

## Abstract

The title compound, C_12_H_15_N_3_O_2_S, adopts an *E* configuration with respect to the C=N bond. The propionate group adopts an anti­periplanar (*ap*) conformation. There are short intra­molecular N—H⋯N and C—H⋯O contacts, forming *S*(5) and *S*(6) ring motifs, respectively. In the crystal, mol­ecules are connected into ribbons extending parallel to [010] by pairs of N—H⋯S inter­actions, forming rings with *R*_2_^2^(8) graph-set motifs, and by pairs of C—H⋯S inter­actions, where rings with the graph-set motif *R*_2_^1^(7) are observed. The O atom of the carbonyl group is disordered over two positions, with a refined occupancy ratio of 0.27 (2):0.73 (2). The studied crystal consisted of two domains.

## Abstract 2

The title compound adopts an *E* configuration with respect to the C=N bond. In the crystal, the mol­ecules are connected by pairs of N—H⋯S inter­actions, forming rings with 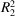 (8) graph-set motifs, and by pairs of C—H⋯S inter­actions, where rings with graph-set motif 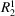 (7) are observed.

## 1. Chemical context

Thio­semicarbazone derivatives have found applications in drug development for the treatment of central nervous system disorders and bacterial infection, as well as analgesic and anti-allergic agents. They are inhibitors of DNA replication and are effective against proteases. This inhibitory activity explains the level of attention given to them in the fight against microbial and parasitic diseases (Mani *et al.*, 2015 ▸). Moreover, thio­semicarbazones have many biological activities, such as anti­parasital (Du *et al.*, 2002 ▸), anti­bacterial, anti­tumour (Papa­georgiou *et al.*, 1997 ▸), anti-African trypanosome (Fatondji *et al.*, 2013 ▸), anti­microbial, sodium channel blocker, anti­malarial, anti­tubercular (Khanye *et al.*, 2011 ▸), anti­viral (Venkatesh *et al.*, 2016 ▸), anti­fungal and locomotor activity (Singh *et al.*, 2011 ▸), and they are used as a cure for leprosy, rheumatism and trypanosomiasis (Parul *et al.*, 2012 ▸). They are also important inter­mediates in organic synthesis, mainly for obtaining heterocyclic rings, such as thia­zolidones, oxa­diazo­les, pyrazolidones and thia­diazo­les (Greenbaum *et al.*, 2004 ▸). Thio­semicarbazones have also received considerable attention in view of their simplicity of preparation and various complexing abilities that can be used in analytical applications (Garg & Jain, 1988 ▸; Casas *et al.*, 2000 ▸). They are well known as *N*,*S*-donors, with a wide range of coordination modes (Lobana *et al.*, 2009 ▸).

In view of such important applications, we herein report the crystal structure determination and Hirshfeld surface analysis of the title thio­semicarbazone derivative, namely, 4-{(1*E*)-1-[(carbamo­thioyl­amino)­imino]­eth­yl}phenyl propano­ate, (I).

## 2. Structural commentary

**Figure 1:**
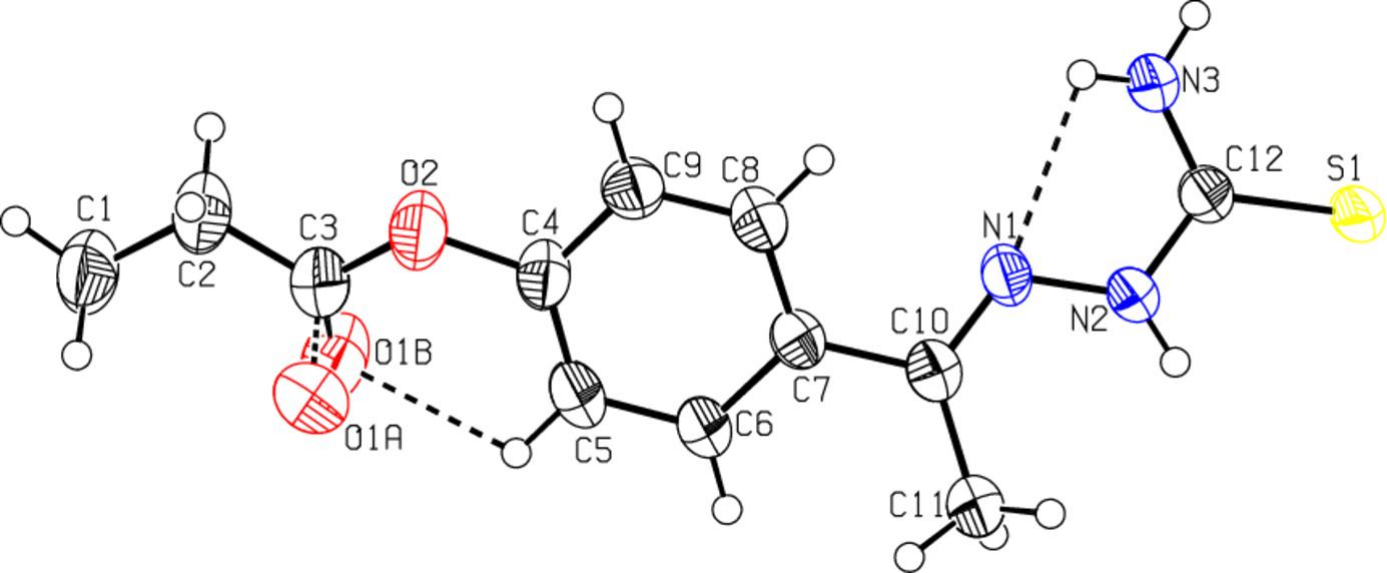
The mol­ecular structure of compound (I), with the atom labelling. Displacement ellipsoids are drawn at the 50% probability level. Intra­molecular contacts are shown as dashed lines.

The mol­ecular structure of compound (I) is shown in Fig. 1 ▸. It adopts an *E* configuration with respect to the C10=N1 bond (Fig. 1 ▸), showing a C10—N1—N2—C12 torsion angle of 175.4 (2)°. The N1—N2—C12—S1 torsion angle of −171.5 (1)° suggests that the thionyl S1 atom is located *trans* to the azomethine N1 atom. The C10=N1 bond length [1.285 (6) Å] is close to that of a formal C=N double bond [1.284 (3) Å; Seena *et al.*, 2006 ▸]. Similarly, the C12=S1 bond length [1.679 (4) Å] is close to that of formal C=S bond [1.685 (3) Å; Jacob & Kurup, 2012 ▸], and the N1—N2 bond length of 1.369 (5) Å is similar to those found in the Cambridge Structural Database (Allen, 2002 ▸) for thio­semicarbazone systems (371 hits, mean N—N distance is 1.374 Å). All other bond lengths and angles are normal and correspond well to those observed in the crystal structures of related semicarbazone and thio­semicarbazone derivatives (Carballo *et al.*, 2014 ▸). 
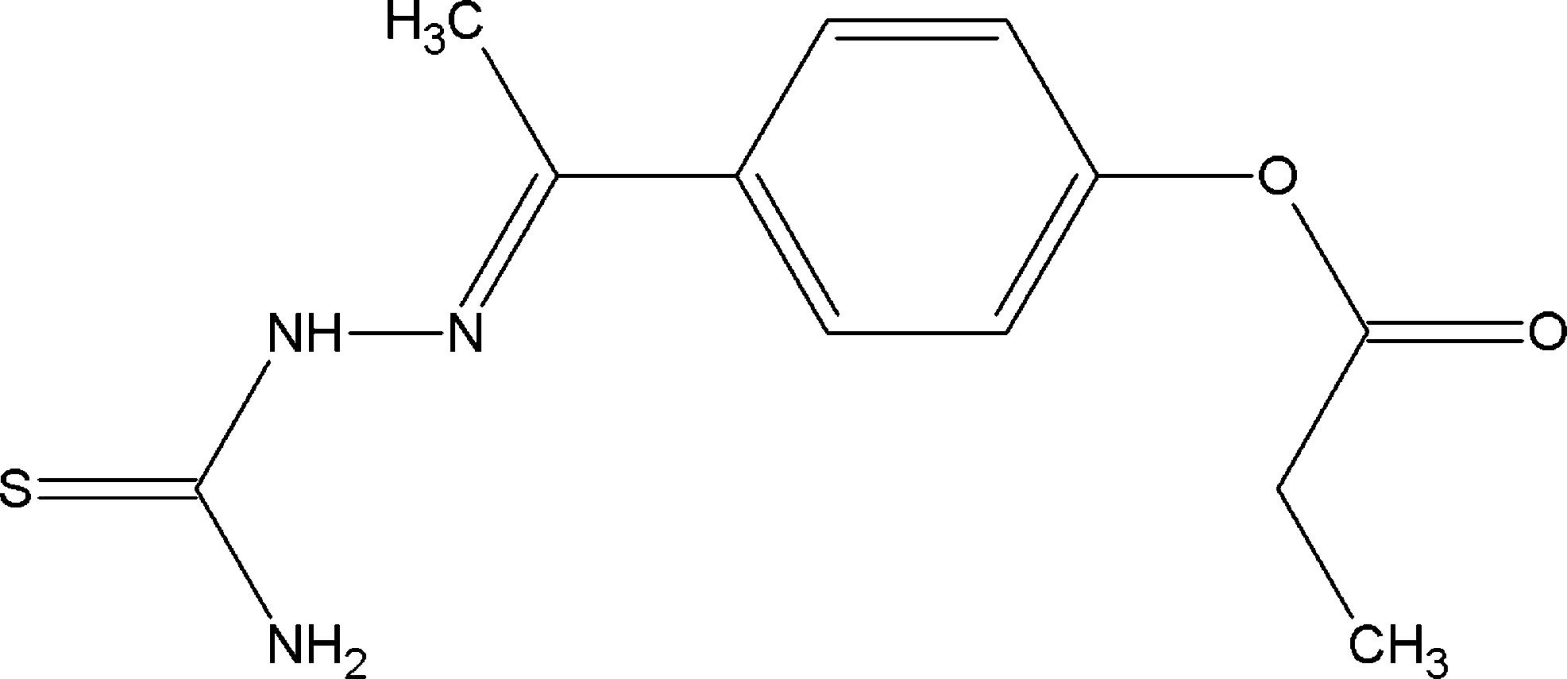


The propionate group adopts an anti­periplanar (*ap*) conformation, as can be seen from the C1—C2—C3—O2 torsion angle of −176.3 (2)°. The semicarbazone unit is nearly planar, showing an N3—C12—N2—N1 torsion angle of 7.4 (2)°. The maximum deviation from the mean plane of the non-H atoms of the C10/C11/C12/N1/N2/N3 fragment is −0.037 (5) Å for the N2 atom. The dihedral angle between this mean plane and the plane of the aromatic ring is 25.3 (1)°. Intra­molecular N—H⋯N and C—H⋯O contacts, forming *S*(5) and *S*(6) ring motifs (Bernstein *et al.* 1995 ▸), respectively, lead to the stabilization of the mol­ecular conformation (Fig. 1 ▸ and Table 1 ▸).

## 3. Supra­molecular features

**Figure 2:**
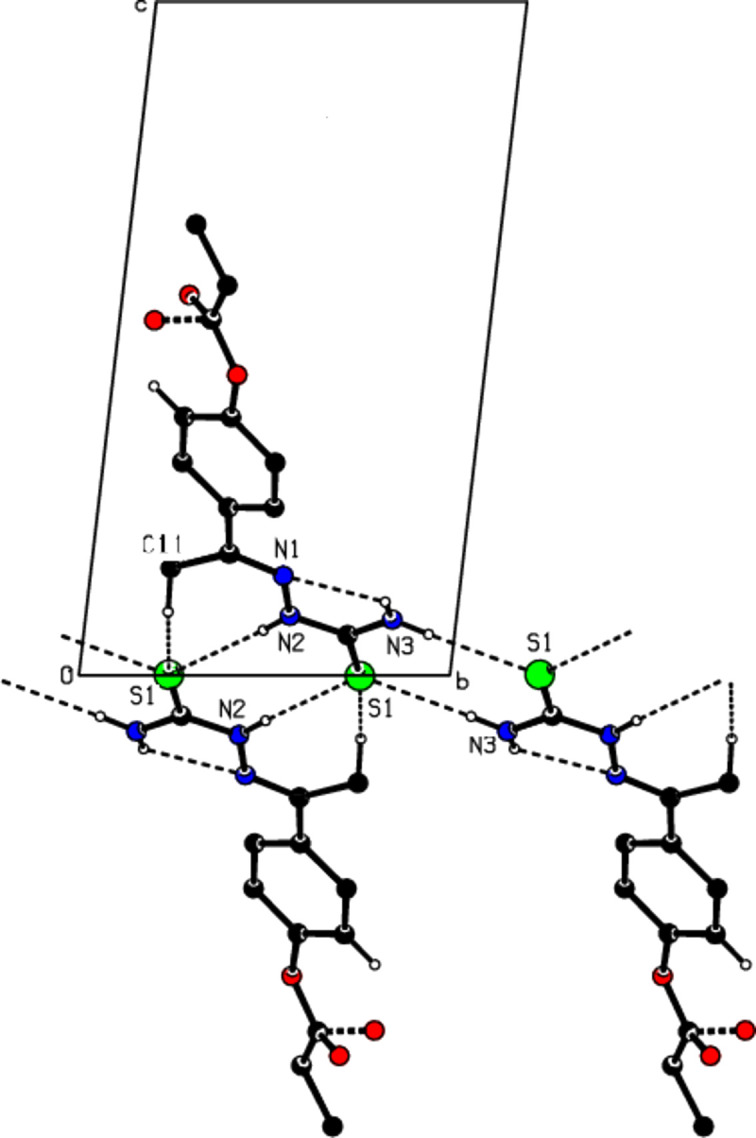
A view along the *a* axis of the crystal structure of (I). Hydrogen bonds are shown as dashed lines and H atoms not involved in hydrogen bonding have been omitted.

Several supra­molecular hydrogen-bonding inter­actions are observed in (I). In the crystal, individual mol­ecules are connected by pairs of N—H⋯S inter­actions, forming ribbons extending parallel to [010], where rings with 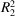 (8) graph-set motifs (Bernstein *et al.*, 1995 ▸) are formed (N2—H2⋯S1 and N3—H3*A*⋯S1), and by pairs of C11—H11*B*⋯S1 inter­actions, where rings with the graph-set motif 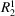 (7) are observed (Fig. 2 ▸). C—H⋯π and π–π inter­molecular inter­actions are not present in the crystal.

## 4. Hirshfeld surface analysis

A recent review by Tiekink and collaborators (Tan *et al.*, 2019 ▸) describes the use and utility of Hirshfeld surface analysis (Spackman & Jayatilaka, 2009 ▸) and the associated two-dimensional fingerprint plots (McKinnon *et al.*, 2007 ▸) for analysis of inter­molecular contacts in crystals. Corresponding calculations were performed with *CrystalExplorer* (Spackman *et al.*, 2021 ▸).

**Figure 3:**
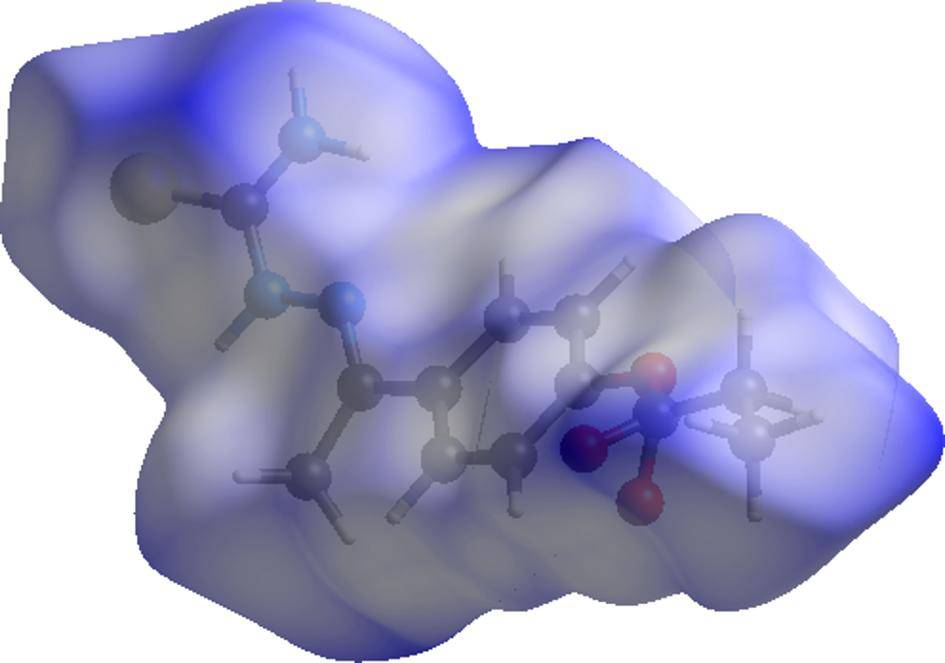
The Hirshfeld surface of (I), mapped over *d*_norm_.

**Figure 4:**
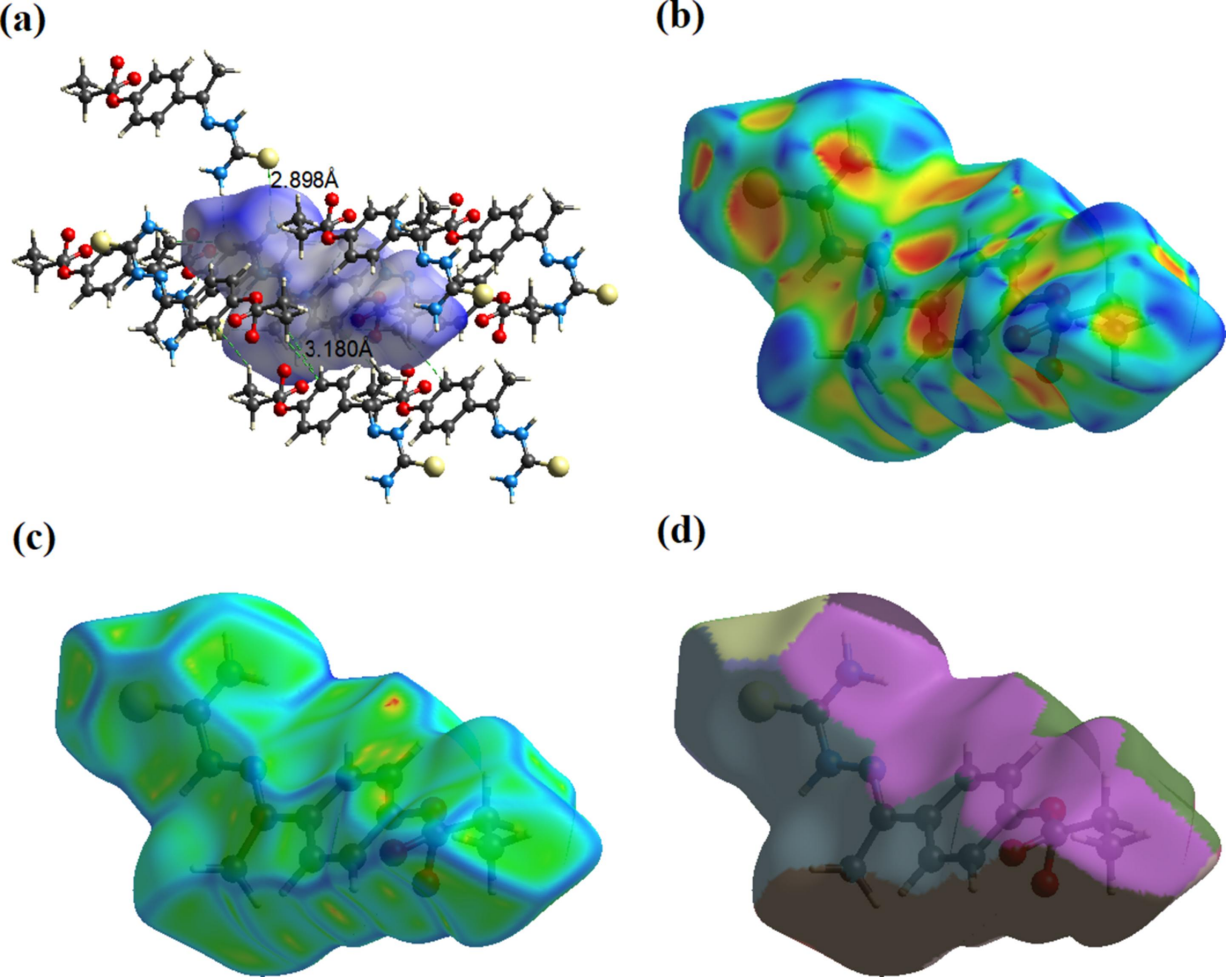
The Hirshfeld surfaces for visualizing the inter­molecular contacts of compound (I): (*a*) *d*_norm_ with various inter­molecular contacts in the crystal, (*b*) shape index, (*c*) curvedness and (*d*) fragment patches.

The Hirshfeld surface of compound (I) mapped over *d*_norm_ is given in Fig. 3 ▸, and the inter­molecular contacts are illustrated in Fig. 4 ▸(*a*). They are colour mapped with the nor­ma­lized contact distance, *d*_norm_, from red (distances shorter than the sum of the van der Waals radii) through white to blue (distances longer than the sum of the van der Waals radii). The *d*_norm_ surface was mapped over a fixed colour scale of −0.469 (red) to 1.632 (blue) for (I), where the red spots indicate the inter­molecular contacts involved in hydrogen-bonding inter­actions. The electrostatic potential was also mapped on the Hirshfeld surface using a STO-3G basis set and the Hartee–Fock level of theory (Spackman *et al.*, 2008 ▸; Jayatilaka *et al.*, 2005 ▸). The presence of inter­actions is indicated by a red and blue colour on the shape-index surface [Fig. 4 ▸(*b*)]. Areas on the Hirshfeld surface with high curvedness tend to divide the surface into contact patches with each neighbouring mol­ecule. The number of inter­acting mol­ecules around a central mol­ecule in the crystal correlates with the curvedness of the Hirshfeld surface [Fig. 4 ▸(*c*)]. The nearest-neighbour coordination environment of a mol­ecule is identified from the colour patches on the Hirshfeld surface depending on their closeness to adjacent mol­ecules [Fig. 4 ▸(*d*)].

**Figure 5:**
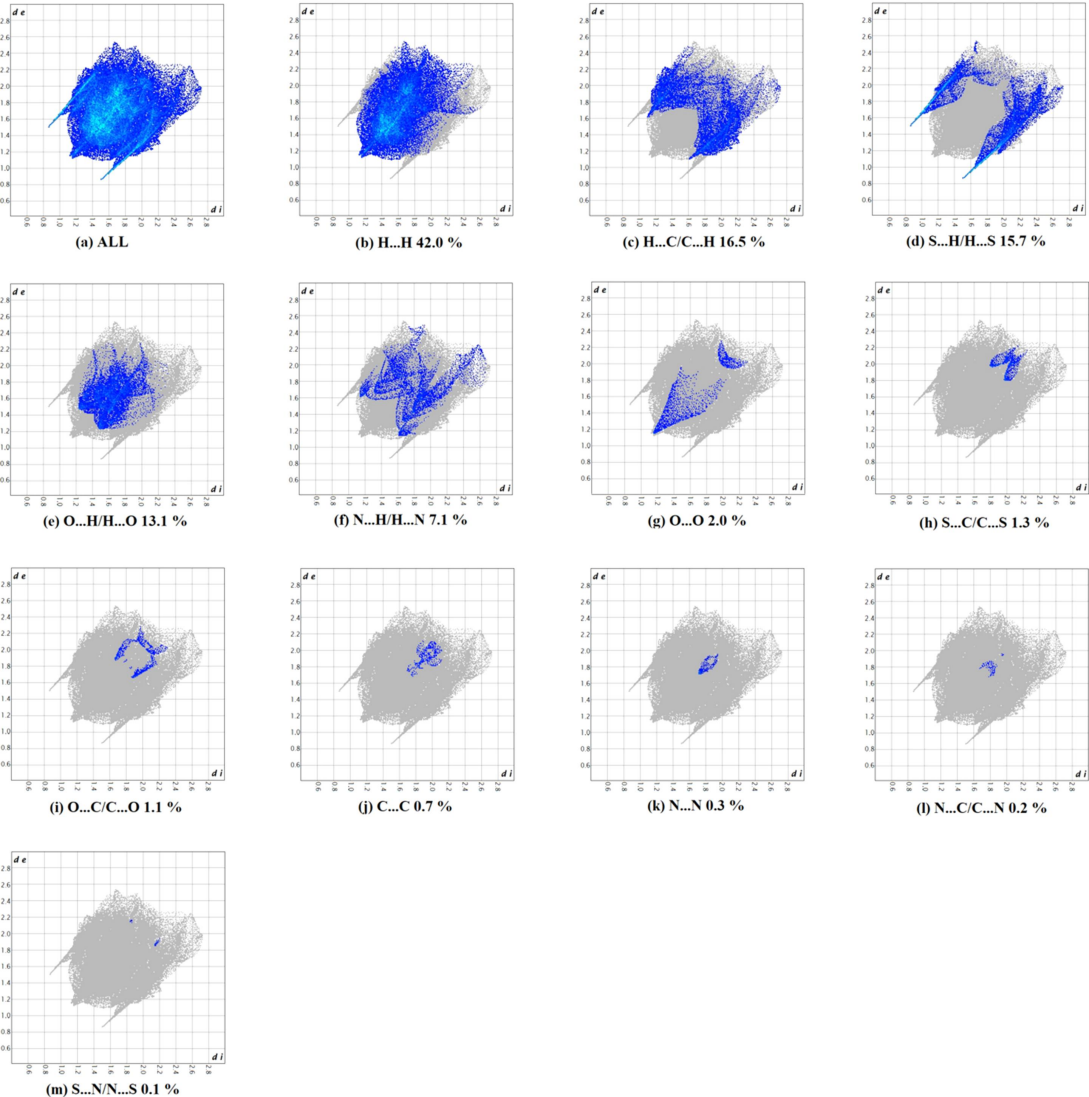
(*a*) The full two-dimensional fingerprint plot for title compound and those delineated into (*b*) H⋯H, (*c*) C⋯H/H⋯C, (*d*) S⋯H/H⋯S, (*e*) O⋯H/H⋯O, (*f*) N⋯H/H⋯N, (*g*) O⋯O, (*h*) S⋯C/C⋯S, (*i*) O⋯C/C⋯O, (*j*) C⋯C, (*k*) N⋯N, (*l*) N⋯C/C⋯N and (*m*) S⋯N/N⋯S contacts.

The fingerprint plots of (I) are given in Fig. 5 ▸. They reveal that the principal inter­molecular contacts are H⋯H contacts with a 42.0% contribution [Fig. 5 ▸(*b*)], followed by H⋯C/C⋯H contacts with a 16.5% contribution (Fig. 5 ▸ *c*), S⋯H/H⋯S with 15.7% [Fig. 5 ▸(*d*)], O⋯·H/H⋯O with 13.1% [Fig. 5 ▸(*e*)] and N⋯H/H⋯N with 7.1% [Fig. 5 ▸(*f*)]. O⋯O contacts with a contribution of 2.0% [Fig. 5 ▸(*g*)], S⋯C/C⋯S with 1.3% [Fig. 5 ▸(*h*)], O⋯C/C⋯O with 1.1% [Fig. 5 ▸(*i*)], C⋯C with 0.7% [Fig. 5 ▸(*j*)], N⋯N with 0.3% [Fig. 5 ▸(*k*)], N⋯C/C⋯N with 0.2% [Fig. 5 ▸(*l*)] and S⋯N/N⋯S with 0.1% [Fig. 5 ▸(*m*)] contribute less to the packing.

## 5. Database survey

Given the inter­est in semi­thio­carbazones owing to their biological potential, it is not surprising that a search of the Cambridge Structural Database (CSD, Version 5.37, last update May 2016; Groom *et al.*, 2016 ▸) revealed almost 100 hits for the CC(H)=NN(H)C(=S)N(H_2_) fragment. The only re­striction in the search was that the heaviest atom was sulfur. In the absence of this restriction, there were nearly 400 hits. All bond lengths and angles are normal and correspond well to those observed in the crystal structures of related semicar­ba­zone and thio­semicarbazone derivatives (Naik & Palenik, 1974 ▸; Wang *et al.*, 2004 ▸; Pelosi *et al.*, 2005 ▸; Yathirajan *et al.*, 2006 ▸; Sarojini *et al.*, 2007 ▸; Reddy *et al.*, 2014 ▸; Carballo *et al.*, 2014 ▸)

## 6. Synthesis and crystallization

To 4-hy­droxy­aceto­phenone (0.5 mol) were added 200 ml of chloro­form under continuous stirring and cooling to 288–293 K. Propanoyl chloride (0.5 mol) was added dropwise to the reaction mixture and stirring continued for another 15 min, when 0.5 mol of potassium carbonate were added slowly. The reaction was continued for another 4 h and was monitored using thin-layer chromatography (TLC). The reaction mass was then washed twice with water (2 × 250 ml). The chloro­form layer was separated and washed with 10 wt% NaOH solution (2 × 250 ml). The aqueous phase was separated, dried with anhydrous sodium sulfate, followed by concentration under reduced pressure using a rotary vacuum system, and cooled before hexane was added.

Thio­semicarbazide (0.91 g, 0.01 mole) was added to 50 ml of an ethano­lic solution of 4-acetyl­phenyl propionate (0.01 mol) with continuous stirring for 4–5 h. The resulting mixture was refluxed at 333 K and the purity of the products, as well as the composition of the reaction mixture, was monitored by TLC using ethyl acetate–hexane (3:7 *v*:*v*). The reaction mixture was cooled to room temperature and the separated product was filtered, dried and finally recrystallized from chloro­form solution, yielding colourless crystals of (I).

## 7. Refinement

Crystal data, data collection and structure refinement details are summarized in Table 2 ▸. Carbon-bound H atoms were placed in calculated positions (C—H = 0.95–0.96 Å) and included in the refinement in the riding-model approximation, with *U*_iso_(H) = 1.5*U*_eq_(C) for methyl H atoms and 1.2*U*_eq_(C) for other H atoms. The N-bound H atoms were located in a difference Fourier map and freely refined. The O1 atom of the carbonyl group was found to be disordered over two positions, with a refined occupancy ratio of 0.73 (2):0.27 (2). The C=O bond length and ADPs were subjected to restraints to yield sensible geometrical parameters. The crystal under investigation consists of two domains. The crystal structure was refined using HKLF5-type data with all reflections of component 1 (including the overlapping ones) resulting in a BASF value of 0.3587 (2).

## Supplementary Material

Crystal structure: contains datablock(s) global, I. DOI: 10.1107/S2056989024003177/wm5713sup1.cif

Structure factors: contains datablock(s) I. DOI: 10.1107/S2056989024003177/wm5713Isup2.hkl

Supporting information file. DOI: 10.1107/S2056989024003177/wm5713Isup3.cml

CCDC reference: 1909895

Additional supporting information:  crystallographic information; 3D view; checkCIF report

## Figures and Tables

**Table 1:** Hydrogen-bond geometry (Å, °)

*D*—H⋯*A*	*D*—H	H⋯*A*	*D*⋯*A*	*D*—H⋯*A*
N3—H3*B*⋯N1	0.80 (3)	2.27 (5)	2.588 (6)	104 (4)
C5—H5⋯O1*B*	0.93	2.32	2.812 (7)	113
N2—H2⋯S1^i^	0.84 (3)	2.69 (3)	3.525 (4)	173 (4)
N3—H3*A*⋯S1^ii^	0.85 (3)	2.55 (3)	3.402 (5)	177 (5)
C11—H11*B*⋯S1^i^	0.96	2.77	3.452 (5)	128

Symmetry codes: (i) 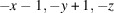 ; (ii) 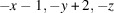 .

**Table 2:** Experimental details

Crystal data	
Chemical formula	C_12_H_15_N_3_O_2_S
*M*_r_	265.33
Crystal system, space group	Triclinic, *P* 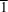
Temperature (K)	296
*a*, *b*, *c* (Å)	5.7700 (1), 8.3069 (2), 14.6243 (5)
α, β, γ (°)	82.891 (2), 87.004 (4), 74.172 (2)
*V* (Å^3^)	669.07 (3)
*Z*	2
Radiation type	Mo *K*α
μ (mm^−1^)	0.24
Crystal size (mm)	0.29 × 0.24 × 0.20

Data collection	
Diffractometer	Bruker D8 VENTURE diffrac­tometer with PHOTON II detector
Absorption correction	Multi-scan (*SADABS*; Krause *et al.*, 2015 ▸)
*T*_min_, *T*_max_	0.723, 0.863
No. of measured, independent and observed [*I* > 2σ(*I*)] reflections	8530, 8530, 6376
(sin θ/λ)_max_ (Å^−1^)	0.595

Refinement	
*R*[*F* ^2^ > 2σ(*F* ^2^)], *wR*(*F* ^2^), *S*	0.065, 0.219, 1.09
No. of reflections	8530
No. of parameters	188
No. of restraints	197
H-atom treatment	H atoms treated by a mixture of independent and constrained refinement
Δρ_max_, Δρ_min_ (e Å^−3^)	0.26, −0.26

Computer programs: *APEX3* (Bruker, 2016 ▸), *SAINT* (Bruker, 2016 ▸), *SHELXT* (Sheldrick, 2015*a* ▸), *SHELXL* (Sheldrick, 2015*b* ▸), *ORTEP-3 for Windows* (Farrugia, 2012 ▸), *Mercury* (Macrae *et al.*, 2020 ▸), *WinGX* (Farrugia, 2012 ▸), *publCIF* (Westrip, 2010 ▸) and *PLATON* (Spek, 2020 ▸).

## supplementary crystallographic information

### Crystal data

**Table:**

C_12_H_15_N_3_O_2_S	*Z* = 2
*M**_r_* = 265.33	*F*(000) = 280
Triclinic, *P*1	*D*_x_ = 1.317 Mg m^−^^3^
*a* = 5.7700 (1) Å	Mo *K*α radiation, λ = 0.71073 Å
*b* = 8.3069 (2) Å	Cell parameters from 8530 reflections
*c* = 14.6243 (5) Å	θ = 1.4–25.0°
α = 82.891 (2)°	µ = 0.24 mm^−^^1^
β = 87.004 (4)°	*T* = 296 K
γ = 74.172 (2)°	Block, colourless
*V* = 669.07 (3) Å^3^	0.29 × 0.24 × 0.20 mm

### Data collection

**Table:**

Bruker D8 VENTURE diffractometer with PHOTON II detector	6376 reflections with *I* > 2σ(*I*)
ω and φ scans	θ_max_ = 25.0°, θ_min_ = 1.4°
Absorption correction: multi-scan (*SADABS*; Krause *et al.*, 2015)	*h* = −6→6
*T*_min_ = 0.723, *T*_max_ = 0.863	*k* = −9→9
8530 measured reflections	*l* = −17→17
8530 independent reflections	

### Refinement

**Table:**

Refinement on *F*^2^	197 restraints
Least-squares matrix: full	Hydrogen site location: mixed
*R*[*F*^2^ > 2σ(*F*^2^)] = 0.065	H atoms treated by a mixture of independent and constrained refinement
*wR*(*F*^2^) = 0.219	*w* = 1/[σ^2^(*F*_o_^2^) + (0.1179*P*)^2^ + 0.1922*P*] where *P* = (*F*_o_^2^ + 2*F*_c_^2^)/3
*S* = 1.09	(Δ/σ)_max_ < 0.001
8530 reflections	Δρ_max_ = 0.26 e Å^−^^3^
188 parameters	Δρ_min_ = −0.26 e Å^−^^3^

### Special details

**Table:**

Geometry. All esds (except the esd in the dihedral angle between two l.s. planes) are estimated using the full covariance matrix. The cell esds are taken into account individually in the estimation of esds in distances, angles and torsion angles; correlations between esds in cell parameters are only used when they are defined by crystal symmetry. An approximate (isotropic) treatment of cell esds is used for estimating esds involving l.s. planes.
Refinement. Refined as a 2-component twin

### Fractional atomic coordinates and isotropic or equivalent isotropic displacement parameters (Å^2^)

**Table:**

	*x*	*y*	*z*	*U*_iso_*/*U*_eq_	Occ. (<1)
C1	1.0534 (11)	0.1766 (9)	0.6695 (4)	0.0748 (18)	
H1A	1.177682	0.206605	0.699122	0.112*	
H1B	1.105508	0.059493	0.660560	0.112*	
H1C	0.909702	0.196951	0.707362	0.112*	
C2	1.0027 (10)	0.2796 (8)	0.5790 (3)	0.0627 (16)	
H2A	0.959090	0.397789	0.588404	0.075*	
H2B	1.148613	0.257722	0.541123	0.075*	
C3	0.8076 (11)	0.2464 (8)	0.5289 (4)	0.0605 (15)	
C4	0.6029 (8)	0.3302 (7)	0.3831 (3)	0.0460 (12)	
C5	0.4929 (9)	0.2042 (7)	0.3839 (3)	0.0553 (14)	
H5	0.527970	0.112943	0.429410	0.066*	
C6	0.3285 (9)	0.2129 (7)	0.3165 (3)	0.0518 (13)	
H6	0.257115	0.125208	0.316087	0.062*	
C7	0.2684 (8)	0.3510 (6)	0.2492 (3)	0.0392 (11)	
C8	0.3902 (8)	0.4745 (7)	0.2493 (3)	0.0443 (12)	
H8	0.359427	0.565770	0.203699	0.053*	
C9	0.5552 (8)	0.4633 (7)	0.3159 (3)	0.0463 (12)	
H9	0.634613	0.546995	0.315112	0.056*	
C10	0.0799 (8)	0.3681 (6)	0.1809 (3)	0.0402 (11)	
C11	0.0131 (9)	0.2144 (7)	0.1596 (3)	0.0538 (14)	
H11A	0.138562	0.115539	0.179483	0.081*	
H11B	−0.007165	0.220584	0.094384	0.081*	
H11C	−0.134948	0.208693	0.191289	0.081*	
C12	−0.3239 (8)	0.7118 (6)	0.0597 (3)	0.0414 (11)	
N1	−0.0217 (6)	0.5204 (5)	0.1479 (2)	0.0407 (10)	
N2	−0.2082 (7)	0.5502 (5)	0.0885 (3)	0.0420 (10)	
N3	−0.2309 (9)	0.8273 (6)	0.0830 (4)	0.0623 (13)	
O2	0.7779 (6)	0.3338 (5)	0.4461 (2)	0.0654 (12)	
S1	−0.5771 (2)	0.75787 (17)	−0.00106 (11)	0.0586 (5)	
O1A	0.829 (7)	0.0938 (10)	0.527 (2)	0.114 (13)	0.27 (2)
O1B	0.6526 (17)	0.1802 (15)	0.5647 (4)	0.084 (4)	0.73 (2)
H2	−0.271 (7)	0.483 (5)	0.067 (3)	0.035 (13)*	
H3A	−0.280 (9)	0.930 (5)	0.061 (3)	0.071 (18)*	
H3B	−0.105 (6)	0.803 (6)	0.109 (3)	0.051 (15)*	

### Atomic displacement parameters (Å^2^)

**Table:**

	*U*^11^	*U*^22^	*U*^33^	*U*^12^	*U*^13^	*U*^23^
C1	0.085 (4)	0.079 (5)	0.060 (3)	−0.012 (4)	−0.028 (3)	−0.013 (3)
C2	0.058 (3)	0.080 (5)	0.052 (3)	−0.020 (3)	−0.016 (3)	−0.001 (3)
C3	0.071 (4)	0.059 (4)	0.053 (3)	−0.020 (3)	−0.021 (3)	0.003 (3)
C4	0.039 (2)	0.059 (3)	0.040 (3)	−0.015 (2)	−0.007 (2)	0.001 (3)
C5	0.058 (3)	0.048 (3)	0.054 (3)	−0.010 (3)	−0.019 (3)	0.012 (3)
C6	0.056 (3)	0.043 (3)	0.055 (3)	−0.015 (3)	−0.021 (2)	0.010 (3)
C7	0.035 (2)	0.037 (3)	0.042 (2)	−0.004 (2)	−0.0033 (19)	−0.002 (2)
C8	0.040 (2)	0.046 (3)	0.043 (3)	−0.008 (2)	−0.004 (2)	0.004 (2)
C9	0.044 (3)	0.047 (3)	0.050 (3)	−0.017 (2)	−0.002 (2)	0.001 (3)
C10	0.038 (2)	0.041 (3)	0.038 (2)	−0.006 (2)	−0.002 (2)	−0.002 (2)
C11	0.062 (3)	0.042 (3)	0.056 (3)	−0.010 (3)	−0.021 (3)	−0.001 (3)
C12	0.040 (2)	0.039 (3)	0.045 (3)	−0.013 (2)	−0.008 (2)	0.004 (2)
N1	0.039 (2)	0.043 (3)	0.038 (2)	−0.0112 (18)	−0.0095 (16)	0.0066 (19)
N2	0.043 (2)	0.033 (2)	0.049 (2)	−0.0095 (19)	−0.0146 (18)	0.005 (2)
N3	0.067 (3)	0.039 (3)	0.081 (3)	−0.017 (3)	−0.044 (3)	0.012 (3)
O2	0.064 (2)	0.084 (3)	0.055 (2)	−0.037 (2)	−0.0220 (18)	0.015 (2)
S1	0.0527 (8)	0.0359 (8)	0.0871 (10)	−0.0128 (6)	−0.0365 (7)	0.0107 (7)
O1A	0.15 (3)	0.056 (13)	0.14 (2)	−0.027 (13)	−0.08 (2)	0.014 (12)
O1B	0.087 (6)	0.125 (9)	0.058 (4)	−0.065 (6)	−0.017 (4)	0.011 (4)

### Geometric parameters (Å, º)

**Table:**

C1—C2	1.480 (7)	C7—C8	1.393 (6)
C1—H1A	0.9600	C7—C10	1.482 (6)
C1—H1B	0.9600	C8—C9	1.375 (6)
C1—H1C	0.9600	C8—H8	0.9300
C2—C3	1.479 (7)	C9—H9	0.9300
C2—H2A	0.9700	C10—N1	1.285 (6)
C2—H2B	0.9700	C10—C11	1.502 (7)
C3—O1B	1.234 (8)	C11—H11A	0.9600
C3—O1A	1.242 (2)	C11—H11B	0.9600
C3—O2	1.327 (6)	C11—H11C	0.9600
C4—C9	1.363 (7)	C12—N3	1.305 (6)
C4—C5	1.362 (7)	C12—N2	1.351 (6)
C4—O2	1.410 (5)	C12—S1	1.679 (4)
C5—C6	1.386 (6)	N1—N2	1.369 (5)
C5—H5	0.9300	N2—H2	0.84 (3)
C6—C7	1.394 (7)	N3—H3A	0.85 (3)
C6—H6	0.9300	N3—H3B	0.80 (3)
			
C2—C1—H1A	109.5	C6—C7—C10	121.7 (4)
C2—C1—H1B	109.5	C9—C8—C7	120.9 (5)
H1A—C1—H1B	109.5	C9—C8—H8	119.6
C2—C1—H1C	109.5	C7—C8—H8	119.6
H1A—C1—H1C	109.5	C4—C9—C8	120.4 (5)
H1B—C1—H1C	109.5	C4—C9—H9	119.8
C3—C2—C1	113.6 (5)	C8—C9—H9	119.8
C3—C2—H2A	108.8	N1—C10—C7	114.6 (4)
C1—C2—H2A	108.8	N1—C10—C11	125.6 (4)
C3—C2—H2B	108.8	C7—C10—C11	119.7 (4)
C1—C2—H2B	108.8	C10—C11—H11A	109.5
H2A—C2—H2B	107.7	C10—C11—H11B	109.5
O1B—C3—O2	121.3 (5)	H11A—C11—H11B	109.5
O1A—C3—O2	113.3 (14)	C10—C11—H11C	109.5
O1B—C3—C2	124.9 (5)	H11A—C11—H11C	109.5
O1A—C3—C2	113.1 (12)	H11B—C11—H11C	109.5
O2—C3—C2	111.6 (5)	N3—C12—N2	116.7 (4)
C9—C4—C5	120.6 (4)	N3—C12—S1	122.8 (4)
C9—C4—O2	114.3 (4)	N2—C12—S1	120.6 (4)
C5—C4—O2	125.1 (5)	C10—N1—N2	119.1 (4)
C4—C5—C6	119.7 (5)	C12—N2—N1	118.2 (4)
C4—C5—H5	120.2	C12—N2—H2	111 (3)
C6—C5—H5	120.2	N1—N2—H2	131 (3)
C5—C6—C7	121.0 (5)	C12—N3—H3A	122 (4)
C5—C6—H6	119.5	C12—N3—H3B	121 (4)
C7—C6—H6	119.5	H3A—N3—H3B	115 (4)
C8—C7—C6	117.4 (4)	C3—O2—C4	124.5 (4)
C8—C7—C10	120.9 (4)		
			
C1—C2—C3—O1B	20.2 (11)	C6—C7—C10—N1	154.4 (4)
C1—C2—C3—O1A	−47 (2)	C8—C7—C10—C11	158.8 (4)
C1—C2—C3—O2	−176.3 (5)	C6—C7—C10—C11	−22.2 (6)
C9—C4—C5—C6	−0.9 (8)	C7—C10—N1—N2	−176.1 (3)
O2—C4—C5—C6	−178.1 (5)	C11—C10—N1—N2	0.3 (7)
C4—C5—C6—C7	−1.8 (8)	N3—C12—N2—N1	7.4 (7)
C5—C6—C7—C8	3.6 (7)	S1—C12—N2—N1	−171.5 (3)
C5—C6—C7—C10	−175.4 (4)	C10—N1—N2—C12	175.4 (4)
C6—C7—C8—C9	−2.8 (7)	O1B—C3—O2—C4	−14.7 (11)
C10—C7—C8—C9	176.3 (4)	O1A—C3—O2—C4	52 (2)
C5—C4—C9—C8	1.8 (8)	C2—C3—O2—C4	−178.9 (5)
O2—C4—C9—C8	179.2 (4)	C9—C4—O2—C3	161.2 (5)
C7—C8—C9—C4	0.2 (7)	C5—C4—O2—C3	−21.5 (8)
C8—C7—C10—N1	−24.6 (6)		

### Hydrogen-bond geometry (Å, º)

**Table:**

*D*—H···*A*	*D*—H	H···*A*	*D*···*A*	*D*—H···*A*
N3—H3*B*···N1	0.80 (3)	2.27 (5)	2.588 (6)	104 (4)
C5—H5···O1*B*	0.93	2.32	2.812 (7)	113
N2—H2···S1^i^	0.84 (3)	2.69 (3)	3.525 (4)	173 (4)
N3—H3*A*···S1^ii^	0.85 (3)	2.55 (3)	3.402 (5)	177 (5)
C11—H11*B*···S1^i^	0.96	2.77	3.452 (5)	128

Symmetry codes: (i) −*x*−1, −*y*+1, −*z*; (ii) −*x*−1, −*y*+2, −*z*.

## References

[bb1] Allen, F. H. (2002). *Acta Cryst.* B**58**, 380–388. 10.1107/s0108768102003890 12037359

[bb2] Bernstein, J., Davis, R. E., Shimoni, L. & Chang, N.-L. (1995). *Angew. Chem. Int. Ed. Engl.* **34**, 1555–1573.

[bb3] Bruker (2016). *APEX2*, *SAINT* and *SADABS*. Bruker AXS Inc., Madison, Wisconsin, USA.

[bb4] Carballo, R., Pino-Cuevas, A. & Vázquez-López, E. M. (2014). *Acta Cryst.* E**70**, o970. 10.1107/S1600536814017255 PMC4186181 25309285

[bb5] Casas, J. S., García-Tasende, M. S. & Sordo, J. (2000). *Coord. Chem. Rev.* **209**, 197–261.

[bb6] Du, X., Guo, C., Hansell, E., Doyle, P. S., Caffrey, C. R., Holler, T. P., McKerrow, J. H. & Cohen, F. E. (2002). *J. Med. Chem.* **45**, 2695–2707. 10.1021/jm010459j 12061873

[bb7] Farrugia, L. J. (2012). *J. Appl. Cryst.* **45**, 849–854.

[bb8] Fatondji, H. R., Kpoviessi, S., Gbaguidi, F., Bero, J., Hannaert, V., Quetin-Leclercq, J., Poupaert, J., Moudachirou, M. & Accrombessi, G. C. (2013). *Med. Chem. Res.* **22**, 2151–2162.

[bb9] Garg, B. S. & Jain, V. K. (1988). *Microchem. J.* **38**, 144–169.

[bb10] Greenbaum, D. C., Mackey, Z., Hansell, E., Doyle, P. S., Gut, J., Caffrey, C. R., Lehrman, J., Rosenthal, P. J., McKerrow, J. H. & Chibale, K. (2004). *J. Med. Chem.* **47**, 3212–3219. 10.1021/jm030549j 15163200

[bb11] Groom, C. R., Bruno, I. J., Lightfoot, M. P. & Ward, S. C. (2016). *Acta Cryst.* B**72**, 171–179. 10.1107/S2052520616003954 PMC4822653 27048719

[bb12] Jacob, J. M. & Kurup, M. R. P. (2012). *Acta Cryst.* E**68**, o836–o837. 10.1107/S1600536812007039 PMC3297895 22412698

[bb13] Jayatilaka, D., Grimwood, D. J., Lee, A., Lemay, A., Russel, A. J., Taylor, C., Wolff, S. K., Cassam-Chenai, P. & Whitton, A. (2005). *TONTO – A System for Computational Chemistry.* Available at: http://hirshfeldsurface.net/.

[bb14] Khanye, S. D., Wan, B., Franzblau, S. G., Gut, J., Rosenthal, P. J., Smith, G. S. & Chibale, K. (2011). *J. Organomet. Chem.* **696**, 3392–3396.

[bb15] Krause, L., Herbst-Irmer, R., Sheldrick, G. M. & Stalke, D. (2015). *J. Appl. Cryst.* **48**, 3–10. 10.1107/S1600576714022985 PMC4453166 26089746

[bb16] Lobana, T. S., Sharma, R., Bawa, G. & Khanna, S. (2009). *Coord. Chem. Rev.* **253**, 977–1055.

[bb17] Macrae, C. F., Sovago, I., Cottrell, S. J., Galek, P. T. A., McCabe, P., Pidcock, E., Platings, M., Shields, G. P., Stevens, J. S., Towler, M. & Wood, P. A. (2020). *J. Appl. Cryst.* **53**, 226–235. 10.1107/S1600576719014092 PMC6998782 32047413

[bb18] Mani, K. A., Viswanathan, V., Narasimhan, S. & Velmurugan, D. (2015). *Acta Cryst.* E**71**, o43–o44. 10.1107/S2056989014026942 PMC4331904 25705498

[bb19] McKinnon, J. J., Jayatilaka, D. & Spackman, M. A. (2007). *Chem. Commun.* pp. 3814–3816. 10.1039/b704980c 18217656

[bb20] Naik, D. V. & Palenik, G. J. (1974). *Acta Cryst.* B**30**, 2396–2401.

[bb21] Papageorgiou, A., Iakovidou, Z., Mourelatos, D., Mioglou, E., Boutis, L., Kotsis, A., Kovala-Demertzi, D., Domopoulou, A., West, D. X. & Dermetzis, M. A. (1997). *Anticancer Res.* **17**, 247–251. 9066660

[bb22] Parul, N., Subhangkar, N. & Arun, M. (2012). *Inter. Res. J. Phar.* **3**, 350–363.

[bb23] Pelosi, G., Pelizzi, C., Belicchi Ferrari, M., Rodríguez-Argüelles, M. C., Vieito, C. & Sanmartín, J. (2005). *Acta Cryst.* C**61**, o589–o592. 10.1107/S0108270105024959 16210765

[bb24] Reddy, M. S., Sarala, Y., Jagadeesh, M., Das, S. K. & Ammireddy, V. R. (2014). *Acta Cryst.* E**70**, o846. 10.1107/S1600536814015098 PMC4158534 25249898

[bb25] Sarojini, B. K., Narayana, B., Bindya, S., Yathirajan, H. S. & Bolte, M. (2007). *Acta Cryst.* E**63**, o2946. 10.1107/S1600536807062599 PMC2915188 21200681

[bb26] Seena, E. B., Manoj, E. & Kurup, M. R. P. (2006). *Acta Cryst.* C**62**, o486–o488. 10.1107/S0108270106014776 16891727

[bb27] Sheldrick, G. M. (2015*a*). *Acta Cryst.* A**71**, 3–8.

[bb28] Sheldrick, G. M. (2015*b*). *Acta Cryst.* C**71**, 3–8.

[bb29] Singh, R., Mishra, P. S. & Mishra, R. (2011). *Inter. J. Pharm Tech. Res.* **3**, 1625–1629.

[bb30] Spackman, M. A. & Jayatilaka, D. (2009). *CrystEngComm*, **11**, 19–32.

[bb31] Spackman, M. A., McKinnon, J. J. & Jayatilaka, D. (2008). *Cryst­EngComm*, **10**, 377–388.

[bb32] Spackman, P. R., Turner, M. J., McKinnon, J. J., Wolff, S. K., Grimwood, D. J., Jayatilaka, D. & Spackman, M. A. (2021). *J. Appl. Cryst.* **54**, 1006–1011. 10.1107/S1600576721002910 PMC8202033 34188619

[bb33] Spek, A. L. (2020). *Acta Cryst.* E**76**, 1–11. 10.1107/S2056989019016244 PMC6944088 31921444

[bb34] Tan, S. L., Jotani, M. M. & Tiekink, E. R. T. (2019). *Acta Cryst.* E**75**, 308–318. 10.1107/S2056989019001129 PMC6399703 30867939

[bb35] Venkatesh, K., Rayam, P., Sekhar, K. P. C. & Mukkanti, K. (2016). *Int. J. Appl. Biol. Pharm. Tech.* **7**, 258–266.

[bb36] Wang, J.-L., Jia, Y.-J. & Yu, M. (2004). *Acta Cryst.* E**60**, o662–o663.

[bb37] Westrip, S. P. (2010). *J. Appl. Cryst.* **43**, 920–925.

[bb38] Yathirajan, H. S., Bindya, S., Narayana, B., Sarojini, B. K. & Bolte, M. (2006). *Acta Cryst.* E**62**, o5925–o5926.

